# Supplementary material for: Journey through the Decades: The Evolution in Treatment and Shared Decision Making for Locally Advanced Rectal Cancer
Source: Cancers (Basel). 2024 Aug 9;16(16):2807. doi: 10.3390/cancers16162807 (PMC11353159; doi:10.3390/cancers16162807)
Supplement: Supplementary file 1 [file cancers-16-02807-s001.zip › cancers-3128774-supplementary.pdf]

Active Surveillance Versus Surgery
Rectal Cancer Decision Tool

This decision tool is for patients with locally advanced rectal cancer who had radiation and chemotherapy and now have no proof of cancer in the rectum. This is called a "clinical complete response." This tool will help explain your treatment options and help you think about which option fits best with your care goals.

What is rectal cancer?

Rectal cancer is an abnormal growth of cells that starts in the rectum. The rectum is the last part of the large intestine that stool moves through before leaving the body.

What is a "clinical complete response"?

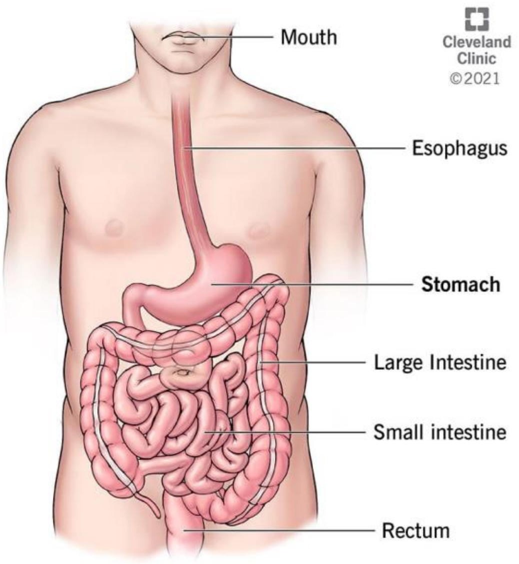

A clinical complete response is when there is no evidence of cancer in the rectum on examination and imaging after getting radiation and chemotherapy.

How is clinical complete response in rectal cancer treated?

In the past, all patients with rectal cancer were treated with surgery. Recent research has found that some patients with a clinical complete response can undergo active surveillance with close follow-up (frequent scans and tests) instead of having surgery[1,2].

Surgery

Surgery involves removing the rectum and the cancer with it. There are different types of surgery, depending on where the cancer is located. After surgery, some patients need a temporary stoma or a permanent stoma. A stoma is an opening on the abdominal wall that a surgeon makes so that stool (poop) can leave the body. This is also called an ostomy, ileostomy, or colostomy.

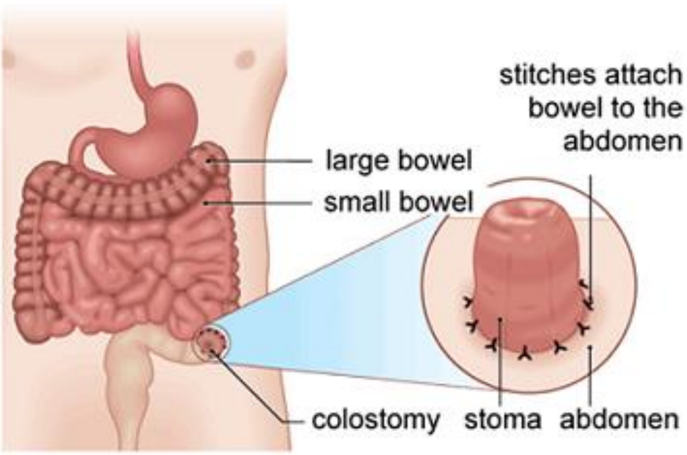

You should talk to your surgeon for more details about surgery.

DRAFT

How long will I need to stay in the hospital after surgery?

- Most patients stay in the hospital for 3-5 days after surgery.

How long will my recovery time be?

- Most patients take up to 6 weeks off work to recover. This will depend on the type of job you have. After surgery, you should not lift more than 10 pounds for 4-6 weeks.

What are some possible short-term issues after surgery?

- **Infection:** About 10-20% of patients will develop an infection, either at their skin, wound or inside the abdomen.[3] Skin infections are treated with antibiotics and wound care. Infections inside the abdomen may need a procedure to drain the infection.

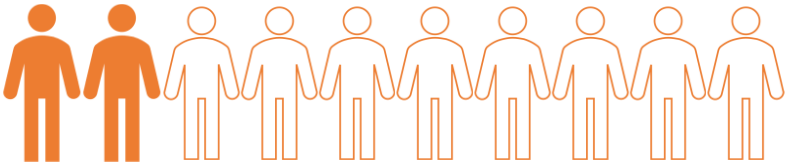

What are some possible long-term issues after surgery?

- **Bowel function:** This is the most common problem after surgery (if you do not have a stoma). It is hard to predict how each patient's bowel function will change, but most patients do notice a change. Up to 75% of patients report leaking stool, a sudden urge to have a bowel movement, and/or more bowel movements through the day[4]. This is called low anterior resection syndrome (LARS). Most people only have minor symptoms while a few have severe symptoms from LARS. Some people have symptoms for a short time, while others have symptoms that are permanent.

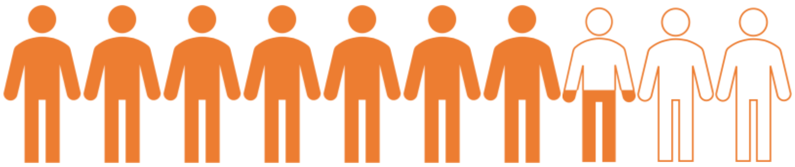

- **Stoma issues:** If you need a stoma, you will have training on how to take care of it. Some common issues include skin irritation (5-25% of patients), dehydration (20% of patients) or problems getting the bag to fit over the stoma[5]. Overall complication rates are between 21-70% [5]. If the stoma is temporary, you will have another surgery 3-6 months later to remove it.

Overall complications: 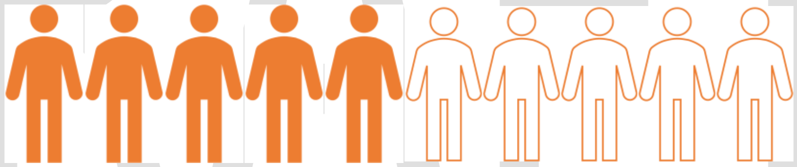

- **Sexual problems:** Between 20-55% of patients report some type of sexual issue after surgery. For men, this is often problems getting or keeping an erection. For women, this is often pain during sex.[6,7] Some sexual problems go away over time or can be treated with medicine.

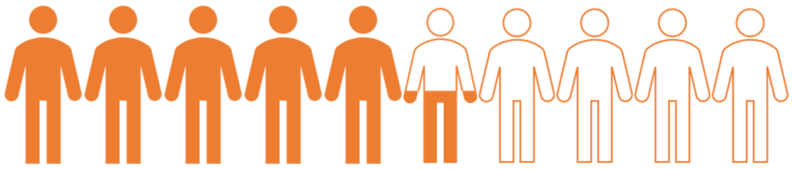

DRAFT

If I get surgery is there a risk of my cancer coming back?

- The three-year risk of your cancer returning is approximately 24% [8].

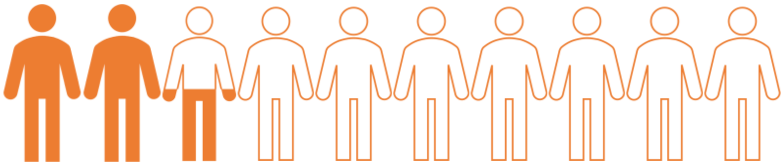

- The three-year risk of your cancer spreading to a different location is 20% [8].

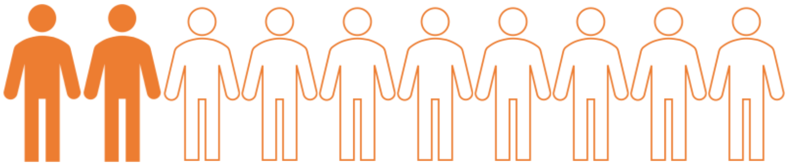

DRAFT

How much will surgery cost?

- Cost can vary widely based on your insurance. Private insurance is billed about $70,000 for the surgery plus hospital stay, but your own out-of-pocket costs (the amount you pay) can vary [9]. You should call your insurance or ask your doctor's office about what your own costs will be.

- If you don't have insurance, you should talk to the hospital or doctor's office about what your costs will be.

- You should also consider time off work and lost wages. Most patients take up to 6 weeks off work to recover.

How often will I have to see a doctor after my surgery?

Regardless of your choice you will be under the care of your colon and rectal surgeon. Our clinic has additional staff which includes nurses and advanced practitioners (NPs and PAs) who are available to you for support or additional appointments if needed. The minimum requirements is:

You will have an appointment 4 weeks after your surgery, then in 6 months and 1 year. You will then undergo yearly visits with your surgeon. A colonoscopy will be performed 1 year after your surgery and then in 3 years and again in 5 years.

Active Surveillance

What is active surveillance?

- If there is <u>no</u> cancer visible on the scans and tests, you can choose to have close follow-up. This is also known as "Watch & Wait". With close follow-up, you will need frequent doctor visits and tests to make sure the cancer has not come back.

How often will I have check-ups and what will they include if I choose active surveillance?

Regardless of your choice you will be under the care of your colon and rectal surgeon. Our clinic has additional staff which includes nurses and advanced practitioners (NPs and PAs) who are available to you for support or additional appointments if needed. The minimum requirements is:

	YEARS 1-2			YEARS 3-5	
	Every 4 months	Every 6 months	Every 12 months	Every 6 months	Every 12 months
Examination					
Flexible Sigmoidoscopy					
MRI					
CT scan					

DRAFT

Definition of terms:
Examination: office visit with one of your treating doctors, can involve a digital rectal exam which is when your physician will use their finger to feel the inside of your rectum for signs of cancer
Flexible sigmoidoscopy: office procedure where your surgeon will look at the inside of your rectum using a small camera.
MRI: imaging test that usually takes 30-60 minutes, but can take up to 2 hours, during which you will need to lie down in a narrow machine without moving.
CT scan: imaging test that usually takes 15-30 minutes.

What is the risk that my cancer will come back?

- 20-25% of patients who had a clinical complete response will have their cancer come back within 5 years, but this is usually caught early [10,11]. This is why patients who choose active surveillance need to have frequent check-ups to see if the cancer has come back.

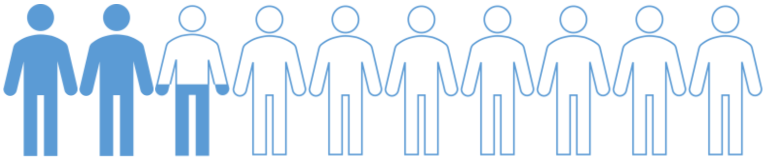

- 80% of patients with a clinical complete response are alive 5 years after diagnosis.[10]
- Some patients may experience anxiety and fear that their cancer will return as well as anxiety around check-ups [12].

What happens if my cancer comes back?

- The goal of the frequent check-ups is to catch the cancer as early as possible if it does come back. Treatment would then include surgery and might include more chemotherapy.

What are the benefits to choosing active surveillance?

- The benefit of choosing active surveillance is that you are able to avoid the risks of surgery described above including infection, poor bowel function, need for a stoma, and sexual problems.

Are there any risks to choosing active surveillance?

- Data are still being collected to answer this question. There are some studies that suggest that survival may be worse if the cancer comes back.[11] Other studies suggest that there is no difference in survival between active surveillance and surgery if the patient has a clinical complete response.[2]

How much does active surveillance cost?

- No studies have been done to show the exact cost of active surveillance and the cost can vary widely based on insurance plan. Studies have shown that active surveillance costs less than surgery overall [13].

- Additional costs to consider are the cost of parking, fees associated with co-payments, costs associated with driving to appointments and time missed from work and lost wages due to appointments.

DRAFT

Overview

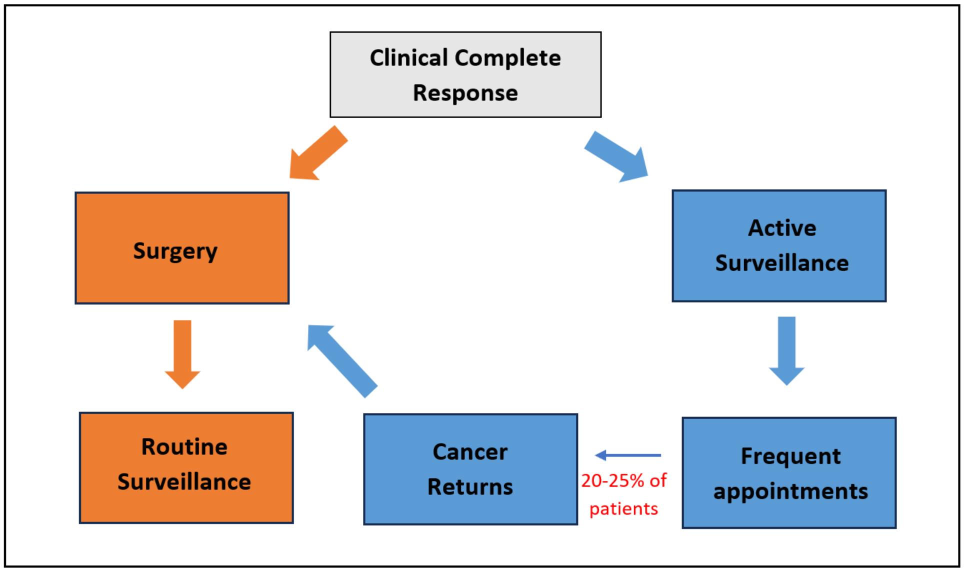

Advantages and Disadvantages of Surgery and Active Surveillance

	Surgery	Active Surveillance
**Advantages**	○ Cancer is removed.<br>○ Specimen can undergo pathologic evaluation to see if there is still cancer present or not.<br>○ Lymph nodes are removed and checked for cancer.<br>○ Remove area of concern which could alleviate anxiety	○ Avoid surgery.<br>○ Avoid possible complications and side effects of surgery.<br>○ No in-patient time spent in the hospital.<br>○ Frequent visits to surveillance areas of concern. If there is recurrence it is usually caught early
**Considerations**		
Recurrence (cancer coming back)	○ Three-year risk of your cancer coming back is 24%<br>Three-year risk of your cancer spreading to a different location is 20%	○ 20-25% risk of your cancer coming back.<br>May require surgery or more chemotherapy if your cancer returns
Cost	○ $70,000 is billed to private insurance. Out of pocket costs (price you pay) can vary.<br>Additional costs: time out of work and lost wages	○ The exact cost billed to private insurance is unknown, but studies have shown that active surveillance costs less than surgery overall.<br>Additional costs: cost of parking, fees associated with co-payments, costs associated with driving to appointments and time missed from work and lost wages due to appointments
**Disadvantages**		
Recovery	○ 3-5 days in the hospital<br>○ 6 weeks of recovery time	
Lifestyle	○ May require temporary or permanent stoma.<br>○ Complications of surgery may impact your lifestyle	○ Frequent doctor visits including examination, flexible sigmoidoscopy, MRI and CT<br>○ May experience anxiety associated with cancer returning
Complications	○ Infection (10-20%)<br>○ Poor bowel function (75%)<br>○ Stoma issues (21-70%)<br>○ Sexual problems (20-55%)	○ Radiation associated with CT scan

DRAFT

Definition of terms:

Examination: office visit with one of your treating doctors, can involve a digital rectal exam which is when your physician will use their finger to feel the inside of your rectum for signs of cancer

Flexible sigmoidoscopy: office procedure where your surgeon will look at the inside of your rectum using a small camera.

MRI: imaging test that usually takes 30-60 minutes, but can take up to 2 hours, during which you will need to lie down in a narrow machine without moving.

CT scan: imaging test that usually takes 15-30 minutes

What factors affect your decision?

Circle all those that apply.

	Yes/No		Comments
I have trouble with transportation to and from appointments	Yes	No	
Fitting in frequent appointments to my daily life can be difficulty	Yes	No	
I currently suffer from incontinence	Yes	No	
I am scared to undergo surgery	Yes	No	
I have anxiety and stress about active surveillance	Yes	No	
I think it is important to avoid having a stoma	Yes	No	
I don't think I would be able to care for myself if I had a stoma	Yes	No	
I am worried that sexual dysfunction would negatively impact my life	Yes	No	

What are you most worried about?
____
____
____
____

What are your next steps?

Please check one:

[ ] I have made a decision.

[ ] I am leaning towards surgery.

[ ] I am leaning towards active surveillance.

[ ] I have not yet made a decision.

DRAFT

References

1. Habr-Gama A, Perez RO, Nadalin W, Sabbaga J, Ribeiro Jr U, Silva e Sousa Jr AH, et al. Operative versus nonoperative treatment for stage 0 distal rectal cancer following chemoradiation therapy: long-term results. Ann Surg. 2004;240(4):711–7.
2. Garcia-Aguilar J, Patil S, Gollub MJ, et al. Organ Preservation in Patients With Rectal Adenocarcinoma Treated With Total Neoadjuvant Therapy. *J Clin Oncol*. 2022;40(23):2546-2556. doi:10.1200/JCO.22.00032
3. Smith RL, Bohl JK, McElearney ST, et al. Wound infection after elective colorectal resection. Ann Surg. 2004;239(5):599-607. doi:10.1097/01.sla.0000124292.21605.99
4. Catherine LC Bryant, Peter J Lunniss, Charles H Knowles, Mohamed A Thaha, Christopher LH Chan, Anterior resection syndrome, The Lancet Oncology, Volume 13, Issue 9, 2012, Pages e403-e408, ISSN 1470-2045, https://doi.org/10.1016/S1470-2045(12)70236-X)
5. Shabbir J, Britton DC. Stoma complications: a literature overview. *Colorectal Dis*. 2010;12(10):958-964. doi:10.1111/j.1463-1318.2009.02006.x
6. Schmidt, C., Bestmann, B., Küchler, T. *et al.* Factors influencing sexual function in patients with rectal cancer. *Int J Impot Res* **17,** 231–238 (2005). https://doi.org/10.1038/sj.ijir.3901276
7. Giglia MD, Stein SL. Overlooked Long-Term Complications of Colorectal Surgery. *Clin Colon Rectal Surg*. 2019;32(3):204-211. doi:10.1055/s-0038-1677027
8. Bahadoer RR, Dijkstra EA, van Etten B, et al. Short-course radiotherapy followed by chemotherapy before total mesorectal excision (TME) versus preoperative chemoradiotherapy, TME, and optional adjuvant chemotherapy in locally advanced rectal cancer (RAPIDO): a randomised, open-label, phase 3 trial [published correction appears in Lancet Oncol. 2021 Feb;22(2):e42. doi: 10.1016/S1470-2045(20)30781-6]. *Lancet Oncol*. 2021;22(1):29-42. doi:10.1016/S1470-2045(20)30555-6
9. Grass F, Merchea A, Mathis KL, Mishra N, Heien H, Sangaralingham LR, Larson DW. Cost drivers of locally advanced rectal cancer treatment-An analysis of a leading healthcare insurer. J Surg Oncol. 2021 Mar;123(4):1023-1029. doi: 10.1002/jso.26390. Epub 2021 Jan 26. PMID: 33497477.
10. van der Valk MJM, Hilling DE, Bastiaannet E, Meershoek-Klein Kranenbarg E, Beets GL, Figueiredo NL, Habr-Gama A, Perez RO, Renehan AG, van de Velde CJH; IWWD Consortium. Long-term outcomes of clinical complete responders after neoadjuvant treatment for rectal cancer in the International Watch & Wait Database (IWWD): an international multicentre registry study. Lancet. 2018 Jun 23;391(10139):2537-2545.
11. Smith JJ, Strombom P, Chow OS et al. Assessment of a Watch-and-Wait Strategy for Rectal Cancer in Patients With a Complete Response After Neoadjuvant Therapy JAMA Oncol. 2019;5(4):e185896.
12. Pennings AJ, Kimman ML, Gielen AHC, Beets GL, Melenhorst J, Breukink SO. Burden of disease experienced by patients following a watch-and-wait policy for locally advanced rectal cancer: A qualitative study. *Colorectal Dis*. 2021;23(11):2870-2878. doi:10.1111/codi.15838
13. Cui CL, Luo WY, Cosman BC, et al. Cost Effectiveness of Watch and Wait Versus Resection in Rectal Cancer Patients with Complete Clinical Response to Neoadjuvant Chemoradiation. *Ann Surg Oncol*. 2022;29(3):1894-1907. doi:10.1245/s10434-021-10576-z
14. Habr-Gama A, Perez RO, Nadalin W, Sabbaga J, Ribeiro Jr U, Silva e Sousa Jr AH, et al. Operative versus nonoperative treatment for stage 0 distal rectal cancer following chemoradiation therapy: long-term results. Ann Surg. 2004;240(4):711–7.
15. Glyn Elwyn, Annette O'Connor, Dawn Stacey, Robert Volk, Adrian Edwards, Angela Coulter, Richard Thomson, Alexandra Barratt, Michael Barry, Steven Bernstein, Phyllis Butow, Aileen Clarke, Vikki Entwistle, Deb Feldman-Stewart, Margaret Holmes-Rovner, Hilary Llewellyn-Thomas, Nora Moumjid, Al Mulley, Cornelia Ruland, Karen Sepucha, Alan Sykes, Tim Whelan, on behalf of the International Patient Decision Aids Standards (IPDAS) Collaboration. Developing a quality criteria framework for patient decision aids: online international Delphi consensus process. British Medical Journal. 2006 Aug 26;333(7565):417.
16. Pennings AJ, Kimman ML, Gielen AHC, Beets GL, Melenhorst J, Breukink SO. Burden of disease experienced by patients following a watch-and-wait policy for locally advanced rectal cancer: A qualitative study. *Colorectal Dis*. 2021;23(11):2870-2878. doi:10.1111/codi.15838

DRAFT
